# Digital Twins in Translational Research and Health Care: An Anthropological Perspective

**DOI:** 10.1002/eahr.70022

**Published:** 2026-05-04

**Authors:** Jennifer K. Wagner

**Affiliations:** ^1^ Assistant professor of law, policy, and engineering and anthropology at Penn State University.

**Keywords:** research ethics, bioethics, digital health, digital twins, dignity, artificial intelligence, precision medicine, privacy

## Abstract

In this essay, I consider the “social life” of digital twins in translational medicine, exploring how the United States is culturally unprepared for the arrival of digital twins at whole person scales. By looking more closely at our anticipated individual and collective interactions with digital twins for biomedical research and health care purposes, I attempt to highlight how our current approach to biomedical innovation could impede the realization of precision medicine rather than enable it. Extensive translational bioethics research—including specifically more deliberate anthroengineering research—is urgently needed so that we can better understand how these dynamic, data intensive, artificial intelligence‐enabled technologies can be responsibly developed, organized, and engaged, and so that we can co‐create the necessary cultural conditions for us all to thrive.

A digital twin (DT) is a high‐fidelity digital representation of a human individual (i.e., a computational model of a person's biological and physiological systems) that is “dynamically updated with data from its physical twin, has a predictive capability, and informs decisions that realize value.”^1^ Excitement and interest in DTs have accelerated rapidly over the past few years, as these and other artificial intelligence (AI)‐enabled technologies are believed to hold tremendous potential for transforming health care and research, as evidenced by a 2024 consensus study report issued by the National Academies of Science, Engineering, and Medicine (NASEM) and a surge of scholarly articles on the topic.^1^, ^2^, ^3^, ^4^ The opportunities and challenges are many, and there is a recognized need to investigate DTs in translational research and health care. A report issued by the Government Accountability Office identified privacy and ethical issues as a key challenge for DTs in medicine, emphasized that existing standards and regulations are likely inadequate, and raised serious policy questions including the need to identify options to address equity issues raised by DTs in health care contexts, steps for mitigating the corresponding privacy and security risks, and standards and regulations appropriate for DTs of individuals in particular.^5^

Scholars have also warned that “existing regulatory principles, oversight methods and ethical frameworks seem out of sync with digital health innovation,”^6^ including but not limited to synthetic data, DTs, and biomedical AI more generally.^7^, ^8^, ^9^ Fortunately, there seems to be broad appreciation that an interdisciplinary approach is essential, as indicated by the Foundations for Digital Twins as Catalyzers of Biomedical Technological Innovation (FDT‐BioTech) program, a joint initiative of the National Institutes of Health, the National Science Foundation, and the US Food and Drug Administration to provide support for interdisciplinary research focused on “synthetic humans” and DTs in research and health care applications.^10^, ^11^ An anthroengineering approach^12^ is overdue. This approach integrates anthropological and engineering theories and methods (including, for example, human‐computer interaction, human‐centered design, ethnographic fieldwork, and participatory methods), to address both short‐term (daily) and long‐term (generational) issues, and could ensure that DTs are culturally situated, socially responsible, and responsive to lived experience. In that spirit, this essay considers the “social life” of DTs in translational medicine, with attention focused on the DTs at the whole human scale.

It must be prefaced that “a complete DT of a whole person has yet to be developed”^13^ and that medical DTs remain largely “in their infancy” or “at the proof‐of‐concept” stage.^4^ Nevertheless, translational bioethics appropriately poses “hard questions” early about a wide range of issues,^14^ with the hope that such deliberations can increase the likelihood of the innovative endeavor being successful and sustainable. With AI‐enabled DTs for translational research and health care, we have a chance to “think ahead” of the technology and be proactive. Remarkable advancements have been made in the scientific pursuit of DTs as envisioned for multiple scales (from the molecular, cellular, and organ scales to whole human and even population scales),^1^ and whole‐body human DTs might not be far away if DTs at those smaller scales (such as the heart or the brain) are successful.

Some scholars have described DTs as “a new paradigm—not merely a method—” for P4 medicine (predictive, preventive, personalized, and participatory).^15^ Designing DTs involves critical decisions regarding “[t]he challenge of embracing or simplifying complexity,”^16^ and the 2024 NASEM report emphasized the importance that DTs be “fit for purpose.”^1^ Specifically when thinking about DTs at the whole human level, it is important to consider how the DTs relate to the individuals from whose data they are generated or for whom they represent as well as how the DTs would be situated and engaged by others within the health care system—in other words, we must consider the “social life” of DTs. (This phrasing is a respectful play on the insightful book by Alondra Nelson, *The Social Life of DNA: Race, Reparations, and Reconciliation after the Genome* [Beacon Press, 2016].)

The creation of a DT for translational medicine requires definitional clarity and consistency, which continues to be lacking.^4^, ^17^, ^18^ DTs are more than just data, but there has been confusion. Stated simply, DTs are distinguishable from individual digital data, composite digital data, and AI‐aided digital data. Individual digital data are discrete records (such as a lab note or wearable sensor stream), whereas composite digital data are digital phenotypes comprising collections or aggregations of individual data lacking dynamic quality. AI‐aided digital data are digital representations, algorithmic transformations of data (such as risk scores, embeddings, and segmentations). In contrast, DTs explicitly are a computational “stand in” for real‐life counterparts, are dynamic and changing with continuous updates from the data connections, and integrate feedback from embedded modeling and “what if” scenario testing. As emphasized in the definition of a DT embraced by NASEM, “the bidirectional interaction between the virtual and the physical is central to the digital twin.”^1^

Even the term “digital twin” is appropriately questioned,^19^ as the language we use and encounter both reflects and shapes cultural meaning.^20^ Alternative names for DTs have included, among others, AI avatar, AI SIM, AI twin, digital duplicate, and digital doppelgänger, which might or might not be suitable for specific contexts.^19^ Regardless of name, DTs can be properly understood as having five distinct components: the individual (patient), the data connection, the patient‐in‐silico model, an AI‐based interface to offer interpretation and decision‐support, and bidirectional synchronization between the individual and patient‐in‐silico model as new data is available.^18^

The development of DTs—their engagement over time, across space, and in different contexts for translational research and health care; and their discontinuation or demise—are all worthy of closer scrutiny. We must think carefully about DTs (across their technological lifespan, which is not inevitably aligned with that of their physical counterpart) regarding (1) how DTs interact with and might affect their human; (2) how DTs interact with health care providers (including physician‐researchers^21^) and might affect physician‐patient relationships and clinical decision‐making;^22^ and (3) even how the DTs interact with others in society (such as biotech/pharma companies, regulatory agencies, insurance providers, or commercial data brokers) and might affect individuals’ broader community ties and status.

The focus of an anthropological inquiry is not so much about how DTs might work technically (or not) but, instead, how people will experience DTs, how values and assumptions will be built and reinforced in these AI systems, and how DTs will be interpreted (e.g., across contexts and cultures, such as urban and rural settings, in integrated health systems or distinct standalone health care facilities, in the United States and elsewhere around the world). It also includes how the meanings people attach to the DTs shape—whether intentionally, knowingly, recklessly, or otherwise—behaviors, relationships, identities, values, and power. We need to consider how we envision our future with DTs, recognizing we might not share a unified vision, and try to take necessary steps now to create the conditions in which that future is achievable.

As other scholars have noted, with DTs we currently have “a chance for bioethics to think ahead of technological development.”^23^ Yet AI advancements are now happening at a dizzying pace, so time is of the essence. Several scholars have been offering important early contributions, detailing numerous thorny issues (such as agency, autonomy, dignity, privacy, security, discrimination, informed consent, inclusion, data integrity, and trust).^13^, ^18^, ^19^, ^23^, ^24^, ^25^, ^26^, ^27^, ^28^, ^29^, ^30^, ^31^, ^32^, ^33^, ^34^, ^35^ For example, as we think about the individual, it would be relevant to examine (1) objective self‐fashioning^36^ and ways in which the (non)existence of a DT or (non)engagement with the DT could alter perceptions of self, health, normality, and identity; and (2) subjective self‐fashioning^37^ and the ways in which the insights drawn from the DT modify connections with segmented patient groups.

Dimensions of dignity, both absolute and contingent, offer a valuable lens and starting point.^30^ As we think about the physician‐patient relationship, close scrutiny of data meaning‐making^38^, ^39^ is needed, as is ethnographic research to uncover chilling effects and friction. This includes patients’ health care seeking or avoidance based on accessibility of DTs as part of care; how to resolve disagreements amongst patient, DT suggestions, and clinical opinions; and determining whether health care systems may retain and engage DTs of former patients. There are also tensions between health care providers and their employer institutions, such as decisions regarding whether, which, and when DTs must, may, or cannot be developed and engaged, which might affect their ability to practice medicine as well as distribution of liabilities. Broader societal implications include justice, equity, stigmatization, and discrimination. One example would be if health insurers deny treatment coverage based on decisions informed by or lacking DT consultation or if nudges of DTs are deployed to influence choices between therapeutic alternatives.^40^

Some promises of DTs for translational medicine, such as de‐risking clinical trials involving human participants^41^, ^42^, ^43^, ^44^ and eliminating animal testing in drug and device development,^45^, ^46^, ^47^ are certainly intriguing, as is the possibility of a world in which we each have a biomedical body double of sorts to accompany us (enabling point‐of‐care consultations and health care decision‐making) or perhaps even stand in for us (enabling remote access health care even in our own physical absence). Yet many of these promises could be foiled before they ever really get started if current ethical and legal frameworks and data practices remain unchanged. There are stubborn obstacles facing the possible integration of DTs for P4 (again, i.e., predictive, preventive, personalized, and participatory) medicine. DTs *might* help predict disease trajectories to enable anticipatory, preventative measures and enable early diagnosis, and DTs *might* help predict a particular individual's health outcomes to specific therapeutic interventions, thereby helping the individual avoid adverse side effects or improve their chances of a better outcome. But these notions face stubborn obstacles and translational gaps.^2^, ^48^

Poor data practices and functionally obsolete data protection frameworks hinder the extent to which an individual might be aware of their DT, get to know their DT, or have the opportunity to engage with their DT—in other words, these practices and frameworks might block participatory research and health care that is increasingly viewed by bioethics experts as critical.^49^, ^50^ For example, one glaring problem is the illusion of de‐identification under the HIPAA Privacy Rule,^51^ which presumes either that a “safe harbor” approach has removed 18 enumerated identifiers or an expert determination certifies that a dataset has been rendered sufficiently de‐identified along with the regulatory exemptions^52^ that allow secondary research of de‐identified data without authorization or specific informed consent. If DTs are technically generated outside of and sequestered from the magical space known as the HIPAA “designated record set” (which might be tempting for proof‐of‐concept research and development of the technologies), the participatory and personalized aspects are dramatically reduced if not eliminated. Moreover, advanced DTs with highly granular, diverse, and multimodal data that are updated frequently (even if not in real‐time) based on “de‐identified” data of an individual are likely to be functionally re‐identifiable.

Depending upon how DTs are conceived and developed, not only might individuals lack an opportunity to contribute knowingly and willingly to the generation of their DTs, but also individuals might not be able to access, interact with, or otherwise control them. This is further concerning because the more hyper‐realistic the DTs are, the greater the chance of re‐identifiability and heightened the societal risks of data harms. DTs and other AI technologies in medicine are both rights‐impacting and safety‐impacting, so it is appropriate to proceed with caution even when enthusiastic about their potential benefits to individuals and populations. It is unfortunate that official US policy has largely abandoned a supportive but cautious approach toward developing safe, ethical, and trustworthy AI^53^, ^54^ with a deregulatory “try‐first” AI strategy, including in high‐stakes contexts like translational medicine.^55^, ^56^, ^57^

Another vexing problem likely to thwart the realization of whole human DTs any time soon (in spite of—or perhaps because of—any early successes with digital twins for smaller scales) is the nature of proprietary interests that shape innovative pursuits. Well‐resourced health care institutions are likely to develop their various DT technologies of varying scales “in house” with a patchwork of biomed/biotech industry partners. Health care institutions and their biomed/biotech industry partners separately and collectively seek competitive edges and impose intellectual property constraints on their innovations, further compounding data fragmentation and interoperability problems. The collaborations and partnerships to develop DT technologies for one specialty area and purpose (such as cardiology) are unlikely to be the same or even aligned with those to develop DT technologies for another specialty area (such as neurology). This undermines the possibility of continuity of care for individuals using whole human DTs, as navigating complex noncompetition agreements, exclusive dealing arrangements, data use agreements, trade secrets, and licensing terms could be unachievable barring external pressures to promote collaboration and coordination for public interests. Notably, interoperability mandates and information blocking prohibitions often do not reach research vendors and activities outside of the electronic health record. Modifications are needed to extend the relevant standards like those found in the 21st Century Cures Act.^58^, ^59^

To say there is a tremendous amount of work to be done to better understand and illuminate the social life of digital twins at whole human scales is a gross understatement. We are culturally unprepared and must prioritize and invest in research to explore ways in which the ethical, legal, and social implications of DTs and other AI technologies can be adaptively and responsibly addressed. Do we need a sunset or expiration date on HIPAA authorizations and informed consent for broad research (analogous to prescription expiration dates) to impel individuals to reassess the contextual circumstances (such as current technological capabilities and purposes; ethical, legal, and regulatory protections; and data benefits and risks in and out of translational medicine) that are central to informed decision‐making? Are DTs for whole humans “quasi‐human subjects” that deserve their own set of research protections aligned with the Common Rule? Should DTs be able to persist in perpetuity? Do we need to reconsider whether 50 years of HIPAA privacy protections following the death of an individual is applicable or adequate for their DT counterparts to be engaged in activities that could benefit others (whether surviving relatives or the broader public)? Should we have “transfer portal” policies to balance interests of health care institutions and patients with respect to resource‐intensive technologies such as DTs so that mobility and portability of DTs is possible for individuals but not without reasonable limitations? How does the (non)existence and (non)engagement of DTs in translational medicine affect trust in the science and the health care system and health equity? The questions seem to be endless, leading to one certainty: there is much ELSI research to be done.^60^, ^61^ Perhaps an anthroengineering approach is just what is needed to help DTs at whole human scales for translational research and health care.
